# Correction: European Cohorts of patients and schools to Advance Response to Epidemics (EuCARE): a cluster randomised interventional and observational study protocol to investigate the relationship between schools and SARS-CoV-2 infection

**DOI:** 10.1186/s12879-023-08065-7

**Published:** 2023-02-16

**Authors:** Sara Raimondi, Sara Gandini, Gibran Horemheb Rubio Quintanares, Ana Abecasis, Pier Luigi Lopalco, Oriana D’Ecclesiis, Susanna Chiocca, Elisa Tomezzoli, Ilaria Cutica, Davide Mazzoni, Nuno Amparo, Marta Pingarilho, Daniela Carmagnola, Claudia Dellavia, Gianvincenzo Zuccotti, Chiara Ronchini, Federica Bellerba, Felix Dewald, Rolf Kaiser, Francesca Incardona, The Eucare WP4

**Affiliations:** 1 Department of Experimental Oncology, IEO, European Institute of Oncology IRCCS, Milan, Italy; 2 Institute of Virology, University Clinics of Cologne, Cologne, Germany; 3 Paul Ehrlich Institut, Langen, Germany; 4 Infectious Diseases Department, Instituto Nacional de Ciencias Médicas y Nutrición Salvador Zubirán, Mexico City, Mexico; 5 Global Health and Tropical Medicine, Institute of Hygiene and Tropical Medicine, New University of Lisbon, Lisbon, Portugal; 6 Department of Biological and Environmental Sciences and Technology, University of Salento, Lecce, Italy; 7 Applied Research Division for Cognitive and Psychological Science, European Institute of Oncology (IEO), IRCCS, Milan, Italy; 8 Department of Oncology and Hemato-Oncology, University of Milan, Milan, Italy; 9 Public Health Clusters’ Public Health Unit of Central Alentejo, Lisbon, Portugal; 10 Department of Biomedical, Surgical and Dental Sciences, University of Milan, Milan, Italy; 11 Pediatric Department, Vittore Buzzi” Children’s Hospital, Milan, Italy; 12 Department of Biomedical and Clinical Science, University of Milan, Milan, Italy; 13 IPRO-InformaPRO S.R.L., Rome, Italy; 14 EuResist Network, Rome, Italy

**Correction** **:** **BMC Infectious Diseases (2023) 23:1** https://doi.org/10.1186/s12879-022-07947-6

Following publication of the original article [1], the authors identified an error in the author name of Claudia Dellavia.

The incorrect author name is: Claudia Dallavia.

The correct author name is: Claudia Dellavia.

The author group has been updated above and the original article [1] has been corrected.

## References

[1] Raimondi S, Gandini S, Rubio Quintanares GH, Abecasis A, Lopalco PL, D’Ecclesiis O, Chiocca S, Tomezzoli E, Cutica I, Mazzoni D, Amparo N, Pingarilho M, Carmagnola D, Dellavia C, Zuccotti G, Ronchini C, Bellerba F, Dewald F, Kaiser R, Incardona F, The Eucare WP4 (2023). European Cohorts of patients and schools to Advance Response to Epidemics (EuCARE): a cluster randomised interventional and observational study protocol to investigate the relationship between schools and SARS-CoV-2 infection. BMC Infect Dis.

